# Inference or Enaction? The Impact of Genre on the Narrative Processing of Other Minds

**DOI:** 10.1371/journal.pone.0114172

**Published:** 2014-12-03

**Authors:** James Carney, Rafael Wlodarski, Robin Dunbar

**Affiliations:** Department of Experimental Psychology, University of Oxford, Oxford, United Kingdom; Max Planck Institute for Human Cognitive and Brain Sciences, Germany

## Abstract

Do narratives shape how humans process other minds or do they presuppose an existing theory of mind? This study experimentally investigated this problem by assessing subject responses to systematic alterations in the genre, levels of intentionality, and linguistic complexity of narratives. It showed that the interaction of genre and intentionality level are crucial in determining how narratives are cognitively processed. Specifically, genres that deployed evolutionarily familiar scenarios (relationship stories) were rated as being higher in quality when levels of intentionality were increased; conversely, stories that lacked evolutionary familiarity (espionage stories) were rated as being lower in quality with increases in intentionality level. Overall, the study showed that narrative is not solely either the origin or the product of our intuitions about other minds; instead, different genres will have different—even opposite—effects on how we understand the mind states of others.

## Introduction

In the last number of years, enactivist accounts of the mind have come to the fore as part of the ‘4E’ revolution in cognitive science [1], [2]. For the most part, this has involved accounts of mind characterised by the eponymous four E's—embodied, embedded, extended and enactive—being pitched against inferentialist approaches that identify cognition as “information processing in the sense of a passive intake of information provided by a ready-made world” [3]. Enactivists, in particular, argue for a model of cognition that emphasises the active role played by perceptual and somatic processes in delivering the world to the subject as ‘pre-interpreted.’ That is, they suggest that the subject does not actively model external reality; instead, its relevant features are perceptually delivered as *already* freighted with the opportunities and affordances that are present in the environment [4]–[7]. To this extent, enactivism can be viewed as a type of materialist phenomenology, in which pre-reflective structures of lived experience are assigned explanatory priority over the symbol manipulation and theory-driven deduction that, for the inferentialist, shape our relationship with the external world [8], [9].

As attempts to delineate some of the fundamental aspects of cognition, these concerns have a justified claim on our attention. Where they gain a particular relevance for the study of *narrative*, however, comes with the issue of other minds. The external world, for human beings, is a world populated by social agents; and inevitably, the disposition of these agents has a crucial bearing on whether a given individual flourishes or not [10]–[13]. Correspondingly, the negotiation and representation of intersubjectivity represents one of the core imperatives of human psychological processing, given that failure to do so will likely incur an evolutionary disadvantage in terms of social or reproductive success [14], [15]. *A fortiori*, this implies that any apparatus capable of mediating the human ability to coordinate with others will itself be subject to selective pressures [16].

It is precisely at this point that narrative enters into the picture. Specifically, both inferentialist and enactivist approaches to cognition identify narrative as a device that is deeply implicated in our engagement with other minds—but from radically opposing perspectives. For the inferentialist, narrative fabulation is the cultural expression of innate Theory of Mind (ToM) abilities, in that it seems to require a pre-existing ability to theorize or simulate the ways in which the mental states of others dispose them to act the way they do [17]–[19]. For the most part, empirical evidence for this position comes from the pathology of conditions like autism, where deficiencies in ToM are linked to problems in understanding and producing narratives [20]–[22]. Against this view, enactivists argue for narrative as the *origin* of our ability to engage with other minds. That is, narratives are volunteered as a cultural repository of explanatory precedents, background knowledge, interactional schemas and dispositional primers that we actively use to understand why others act the way they do [23]–[28]. This position meets the challenge of deficiencies in narrative comprehension and production on the part of autistic subjects by reversing the causal direction—that is, autism traits are cited as the *consequence* of being unable to process narratives rather than the *cause* [29], [30]. The result is that we have two diametrically opposed positions concerning the role played by narrative in social cognition, and no conclusive argument for resolving which the correct one is. How might we break this deadlock?

The current study attempts to resolve this dilemma experimentally by examining reader responses to fictional narratives that systematically vary along several variables, namely linguistic complexity, genre and mentalizing level. Here, ‘mentalizing level’ refers to the amount of entrained belief states of the form ‘*A* believes that *B* thinks that *C* doubts … that *X* is the case.’ As these variables are both central to narrative and implicated in the processing of other mind states, by manipulating them it becomes possible to makes (and test) predictions concerning the relative efficacy of inferential and enactive models of narrative cognition.

The key area in which these two cognitive accounts yield different predictions relates to the role of *cognitive load* [31]–[33] in the processing of narrative. On the inferentialist side, previous research predicts that increasing cognitive load in the form of mentalizing should positively influence subjective ratings of narrative quality, irrespective of the level of linguistic complexity or literary genre of the narrative in question. This is because ToM is posited as a relatively discrete competence which relies upon dedicated cognitive mechanisms [34], [35], and thus should not necessarily be affected by higher cognitive processing requirements in other domains. The link between subjective ratings of narrative quality and high levels of mentalizing comes from the claims of Dunbar [17] and Zunshine [18], who argue that literary quality consists (at least partially) in the ability of a text to push readers to their cognitive limits. This yields our first testable hypothesis:

**Hypothesis 1**: Higher levels of mentalizing in a narrative should yield higher subjective ratings of quality, independent of genre type or linguistic complexity.

Against this, the enactivist view predicts that increasing cognitive load in the form of mentalizing should only yield an increase in subjective ratings of quality when the narrative genre exploits familiar ‘folk-psychological’ scenarios [30]. Here, the reasoning is that such genres are sufficiently similar to the typical ways in which we reason about other minds to therefore activate cognitive schemas that reduce cognitive load and leave capacity free for engaging with innovations introduced by high mentalizing levels. Conversely, when an unfamiliar genre is coupled with high mentalizing levels, the resulting high levels of cognitive load should lead to lower ratings of narrative quality, due to the overly novel stimuli inhibiting hedonic engagement [36], [37]. This gives our second hypothesis:

**Hypothesis 2**: Increased levels of mentalizing will only increase ratings of narrative quality when the genre exploits familiar folk-psychological scenarios.

The final hypothesis concerns the role of language in narrative appreciation. In one sense, this is the default position of disciplines like literary studies, given the persistent identification of linguistic innovation as a necessary (but not always sufficient) component of the literary aesthetic [38], [39]. Certainly, it would be remiss *not* to test for the effects of linguistic complexity in any assessment of reaction to narrative materials. Given that (to the authors' knowledge) there is no attested relationship between linguistic complexity and mentalizing level or literary genre, our third hypothesis therefore runs as follows:

**Hypothesis 3**: Linguistic complexity will increase ratings of narrative quality independently of the effects of mentalizing level and genre.

These, then, are the three hypotheses to be tested in this study. To be clear from the outset, it is not required that these hypotheses should (or for that matter, should not) be mutually exclusive: it is possible, for instance, that each hypothesis might have a scope-restricted validity, or that one hypothesis could be collapsed into another. Nevertheless, the outlined programme of experimentation enables us to make a decisive intervention in the debate concerning the modes of cognition deployed in the reception of narrative.

## Materials and Methods

### Participants

Participants for the one-hour study were recruited via email, using mailing lists available in the Department of Experimental Psychology at the University of Oxford. Of the 96 participants who participated in the study, all completed the questions required for further data analysis. This cohort had a mean age of 21.8 (*SD*  = 4.21), with male participants making up 29% of the sample, and 98% of respondents being undergraduate or postgraduate students. Participants were paid £10 for their time. Ethics approval was provided by CUREC, the Central University Research Ethics Committee at Oxford (no. MSD-IDREC-C1-2013-59). As approved by CUREC, written consent was taken at the start of the study by asking participants to check a series of questions that indicated their understanding of the study and willingness to participate in it.

### Procedure

The survey was administered in a computer lab in ten one-hour timeslots, each of which accommodated approximately ten participants. On arrival, participants were assigned to a private PC computer terminal, where, after the consent form, study materials were presented and responses collected using online survey software. Participants were firstly asked to complete basic demographic data. Participants then read two short stories of approximately 1,000 words, each in one of two different genres. On completion of each story, they answered comprehension questions relating to each story, and filled a battery of questions that measured participant responses to each story along several transport-related dimensions. All replies were anonymous and no personal data were retained.

### Narrative materials

The short story materials were specifically constructed for this experiment by the lead author to allow maximum scope for manipulation. These materials comprised eight different narratives that systematically varied along three dimensions—namely, mentalizing level, text complexity and genre. Starting with the latter, the two genres adopted—‘espionage’ and ‘relationship’ fiction—were chosen so as to offer a respective contrast and identity with folk-psychological scripts. That is, a story detailing everyday relationships would ostensibly incur less cognitive load than one dealing with the relatively arcane interactions of intelligence agencies and spies, due to its more predictable subject matter [40], [41]. Within each genre, there was a core plot that was adjusted as necessary with respect to the other independent variables. In the relationship condition, the core plot centred on an unsatisfied wife who, in concert with her lover, contrives for her husband to be seduced by an escort so as to secure a guilt-free divorce. The espionage condition focused on the attempt by a senior officer in MI6 to use a mole to frame the head of British operations in the HVA (the East German intelligence agency) as an MI6 double-agent with a view to neutralising him as a threat. The text of all eight narratives can be found in the supplementary materials online.

The second independent variable, mentalizing level, was manipulated by augmenting the core plot with a further set of circumstances that involved two more entrained mental states. This meant that each genre contained a narrative with either ‘level three’ intentionality (‘*A* believes that *B* thinks that *C* is certain of *X*’) or ‘level five’ intentionality (‘*A* believes that *B* thinks that *C* is certain that *D* hopes that *E* knows *X*’). Thus, in the relationship narrative, the husband of the cheating wife is now secretly gay, and has paid her lover to seduce her so she’ll voluntarily leave him without his having to disclose that their marriage is a sham. However, as *she* wants the husband to leave *her*, he must then play along with the ruse involving the escort that she contrives to bring this about. Equivalently, in the espionage condition, the attempt to frame the HVA officer using the mole is used as a smokescreen for a politically sensitive operation to draw out a CIA informant operating at the highest level of MI6. The point to retain is that the intentional structure of both stories is, relative to level, exactly the same; so, in purely mentalizing terms, they should impose equivalent cognitive load.

The final independent variable we manipulated was linguistic complexity. While it would be incorrect to conflate narrative quality with the sophistication of the language in which it is written (after all, narratives need not be verbal), there remain grounds for expecting that linguistic complexity will have a bearing on audience engagement. Certainly, relevance-theoretic approaches to language processing suggest that, up to a certain point, increasing the cognitive load associated with a communicative act will engender higher levels of cognitive engagement as listeners attempt to infer the pragmatic relevance of the higher degree of complexity [42]–[44]—a claim that has already been developed in the context of literary style [45], [46]. For this reason, each narrative was also written in two linguistic variants, with each variant corresponding to high and low linguistic complexity. Though several objective measures of 'linguistic complexity' exist, there is little consensus concerning which metric best matches intuitive notions of complexity [47]. In this study we produced both ‘high’ and ‘low’ linguistic complexity story versions, with complexity assessed using both subjective ratings (obtained during piloting) and the Flesch reading ease scale. This allowed for the stories to be written at gross levels of ‘simple’ and ‘complex’ as determined by the Flesch scale, while also reflecting the subjective criteria that enter into ratings of quality. The end result of manipulating all three variable combinations was eight narratives that systematically varied in terms of genre, mentalizing level and linguistic complexity.

### Reader assessment materials

Each reader was pseudo-randomly assigned to be presented with a sample of two narratives, one from the espionage genre and the other from the relationship genre. These narratives were always both of the same level of linguistic complexity, but differed in mentalizing levels. Reader response to each individual narrative was assessed by asking various questions related to subjective ratings of literary quality, as well as questions about general engagement with the text. Participants rated their agreement with each item using Likert-type scales which ranged from 1 to 7 (1 =  not at all, 7 =  very much).

To extract questions which related most closely to the variable of interest in this study—namely subjective ratings of 'literary quality’—answers to all these items were subjected to a factor analysis. Visual inspection of the scree plot from the factor analysis suggested that questions loaded onto four main factors accounting for 57.5% of the variance, which were then extracted (with varimax rotation) for further examination. The second extracted factor related most closely to ratings of literary quality, and included three questions which co-varied to a high degree (all factor weights >0.688): “*The narrative was well-written*,” “*The language of the narrative was very literary*,” and “*This story is as an example of literary fiction (fiction that is extremely well written)*.” Responses to these three items were then averaged and used as the main dependent variable, i.e. the ‘literary rating’, in further analyses.

### Statistical Analyses

As each participant rated two different stimuli, multilevel modelling techniques were used to analyse the data due to the non-independence of these within-subject ratings—ratings of the different stories were treated as Level 1 units of analysis, with participants acting as Level 2 units of analysis. A 2×2×2 Mixed Linear Model was created with participant ratings of the different stories treated as random factors (with random slopes and intercepts). In this model, story genre was treated as a within-subject fixed factor (two levels), mentalizing levels as a within-subject fixed factor (two levels), and language complexity as a between-subject fixed factor (two levels). This model included tests for main fixed effects as well as all two-way and three-way interaction effects, with all analyses carried out in SPSS (version 22.0).

## Results

Results from the Mixed Linear Model analysis showed that was a main effect of story genre on literary ratings (*F*(1,96) = 38.582, *p*<.001), with no significant main effects of language complexity (*F*(1,96) = 1.830, *p* = .179) or mentalizing levels (*F*(1,96) = 0.488, *p* = .487). Furthermore, the analysis suggested the presence of a two-way interaction effect between genre and mentalizing levels (*F*(1,96) = 5.982, *p* = .016) and a three-way interaction between genre, mentalizing levels and language complexity (*F*(1,96) = 13.687, *p*<.001), with the other two interaction effects not reaching statistical significance (*p*s>.180).

**Figure 1:**
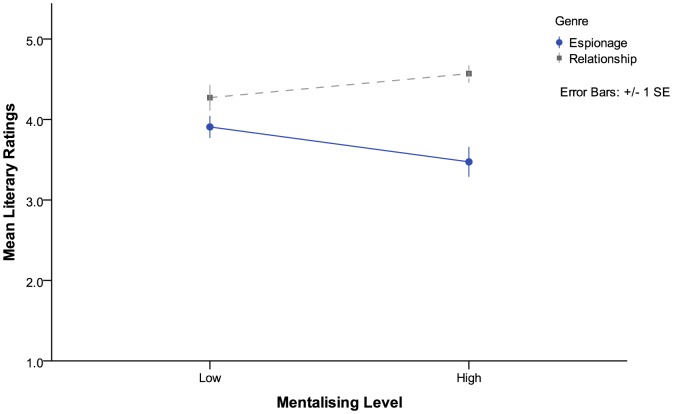
Interaction effect of genre and mentalizing levels on participant literary ratings.

This analysis suggests that, overall, stories which were written in the espionage genre were rated as lower literary quality (*M* = 3.68, *SE*  = .10) than stories written in the relationship genre (*M*  = 4.41, *SE*  = .10). The interaction effect between genre and mentalizing suggested that when it came to espionage stories, stories with high levels of mentalizing were seen as less literary (*M*  = 3.34, *SE*  = .14) than stories with low levels of mentalizing (*M*  = 3.93, *SE*  = .15), while for relationship texts the opposite was true, with relationship texts with high levels of mentalizing seen as more literary (*M*  = 4.58, *SE*  = .15) than those with low levels of mentalizing (*M*  = 4.25, *SE*  = .14) (see Figure 1).

**Figure 2:**
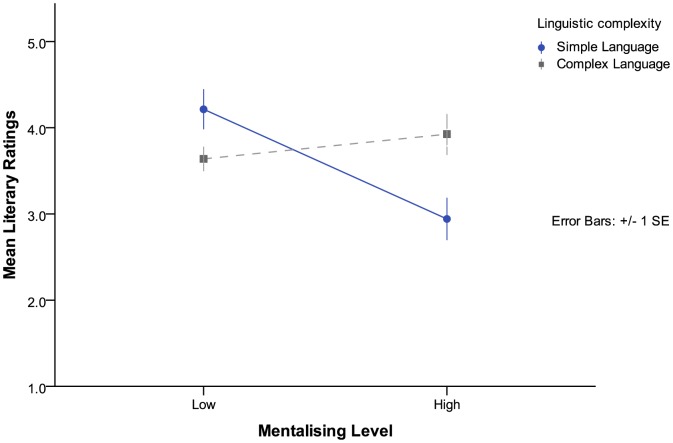
Espionage Genre—Interaction effect of language complexity and mentalizing levels on participant literary ratings.

The significant three-way interaction effect was de-composed using additional MLM models which separately examined, within each of the two genres, the two-way interactions between mentalizing levels and language complexity on literary ratings. Within the espionage genre, a significant main effect was found for mentalizing levels (*F*(1,91)  = 4.90, *p*  = .029) but not for language complexity (*F*(1,91)  = 0.842, *p*  = .361), with a significant two-way interaction mediating between mentalizing levels and language complexity (*F*(1,91)  = 12.28, *p*<.001). This result suggests that when it came to rating the literary quality of the espionage text, texts with higher levels of mentalizing were generally rated as being of lower literary quality (high mentalizing *M*  = 3.43, *SE*  = .15, low mentalizing *M*  = 3.93, *SE*  = .16). The interaction effect further showed that if the text had simple language then it was rated as having much lower literary quality when it involved high levels of mentalizing (*M*  = 2.94, *SE*  = .23) than if it involved low levels of mentalizing (*M*  = 4.21, *SE*  = .24); alternatively, if the text used complex language there was little difference in literary ratings between a text with low levels of mentalizing (*M*  = 3.64, *SE*  = .22) and high levels of mentalizing (*M*  = 3.93, *SE*  = .21) (see Figure 2).

**Figure 3:**
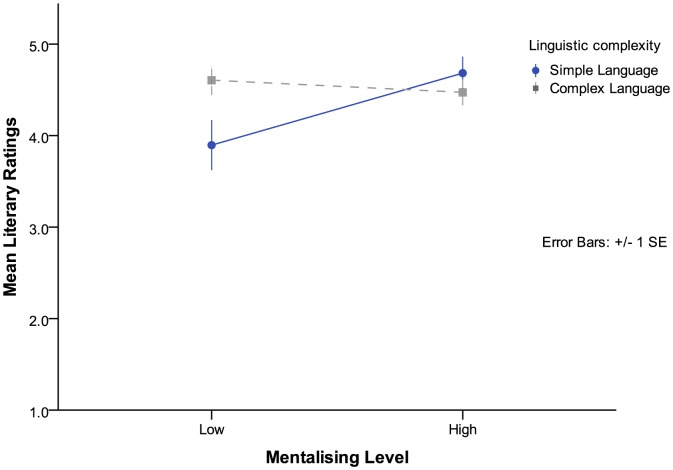
Relationship Genre—Interaction effect of language complexity and mentalizing levels on participant literary ratings.

In the relationship genre, there were no main effects for either mentalizing levels (*F*(1,91) = 2.79, *p* = .098) or language complexity (*F*(1,92) = 1.63, *p* = .206); however a significant two-way interaction was again found between mentalizing levels and language complexity (*F*(1,91) = 5.52, *p* = .021). This result suggests the opposite trend to that for the espionage texts, insofar as when a story contained simple language it was rated as having much higher literary quality if it also had high levels of mentalizing (*M*  = 4.68, *SE*  = .21) than when it had low levels of mentalizing (*M*  = 3.90, *SE*  = .20). However, when the story contained complex language, there was no difference in ratings of literariness between high (*M*  = 4.47, *SE*  = .20) and low (*M*  = 4.61, *SE*  = .18) mentalizing texts (see Figure 3).

## Discussion

We have shown that all three independent variables examined (genre, linguistic complexity and mentalizing levels) combined to affect readers’ perceptions of literary quality. It was found that, within the espionage genre, only among texts which used simple language were ratings of literary quality reduced by the introduction of higher levels of mentalizing complexity. Conversely, within the relationship genre, only among texts that used simple language did additional levels of mentalizing complexity increase subjective ratings of literary quality.

Broadly speaking, this means that our results support the enactivist position (hypothesis 2), to the extent that combining high levels of mentalizing with a genre that ostensibly imposes a heavier cognitive load leads to reduced ratings of narrative quality. However, the fact that the different genres attract different responses suggests that the ‘strong’ enactivist position—the claim that human beings *never* use ToM in processing fictional narrative [25], [48]—is likely false. This has the converse implication that elements of the inferentialist position (hypothesis 1) are conditionally supported, given the fact that, when the genre imposes ostensibly low cognitive load, ratings of quality increase. In terms of hypothesis 3—the claim that ratings of quality will increase with levels of linguistic complexity—results show that, while mean ratings of literariness are higher in the complex language condition for both genres, these differences are not statistically significant. Thus, responses are consistent with hypothesis 3 being either true or false. Given the extent to which traditional literary studies emphasise the role of semantic and linguistic innovations in readers’ engagement with texts, the equivocal nature of this result is somewhat surprising. It may be that this result can be explained as the consequence of averaging responses across all participants, and that a more fine-grained partitioning of subjects in terms of reading experience and verbal-linguistic ability should resolve the issue one way or the other.

More generally, the key question is how we explain our results. For us, the most reasonable account is offered by the view that schematic cognition is activated by familiar genres and the relevant schemas free up cognitive resources by reducing the need to process novel stimuli [49]. In this, we reflect the large literature on schema theory and narrative, which has consistently attested to the role played by background knowledge in narrative understanding [40], [41], [50], [51]. Nevertheless, this only serves to place the question at a further remove, in that a thorough explanation should also account for the provenance of the schemata in question. The most parsimonious explanation in this regard is simple familiarity: people experience espionage narratives as imposing a higher cognitive load because they are exposed to fewer instances of espionage fiction, and thus lack schema-driven expectations. However, this response is problematized by the fact that, among the total sales of genre fiction in the UK, crime and thrillers (of which espionage fiction forms a part) represent up to 59% of the market [52]. Therefore, simple familiarity alone does not seem to be enough to account for the observed variation, and we need to look elsewhere for our explanation.

On our view, the most effective expedient here is to focus on the *content* of the narratives. Though both the espionage and relationship stories deal with interpersonal conflicts and alliances, they do not do so in relevantly similar contexts. For the fact is, while very few people will ever be exposed to the machinations of cold war intelligence agencies, knowledge of sexual relationships is a matter of vital concern to virtually all post-pubescent human beings. The result is that there may well exist evolutionarily innate pre-reflective preferences and strategies that attach to the domain of intersexual relations [53], [54]. As might be expected, monitoring the possibility of infidelity would likely be one such strategy in both sexes’ behavioural repertoires. Certainly, while it is impossible to gain data on extra-pair couplings in the Palaeolithic, it is estimated that, in contemporary North American populations, rates of extramarital sex can be up to 25% in heterosexual marriages—with the propensity to infidelity only marginally favouring men [55]. Given the relative costs of mistaken paternity (for men) or non-exclusive access to paternal resources (for women), the principal outcome of this is that there should be a persistent anxiety attaching to the issue of infidelity, which selectively (and unconsciously) foregrounds stimuli that are relevantly associated with it while dismissing those that are not [56]–[58]. Correspondingly, if schematic thinking is responsible for the low cognitive load of the relationship genre, it may well be a product of evolutionary pressures that have selected for human beings showing hyper-vigilance for cues that signal infidelity—even when these cues only attach to third parties [59], [60]. The end result is that narratives featuring infidelity should engage attention without overly impinging on cognitive resources.

Bringing this back to our results, what emerges is that the opposite effects of high mentalizing in different genres is likely the result of a cognitive load issue. On the one hand, the pre-reflective nature of our thinking about relationships frees up processing power that can then be deployed on theory of mind tasks—and from this, follows the positive response to high levels of mentalizing. On the other hand, the espionage narratives are likely to have far less intuitive traction for most readers, so proportionately fewer resources are available for tracking mental states. Correspondingly, high levels of mentalizing in the latter scenario are likely to push many readers above their cognitive ceiling, resulting in lower ratings of quality. Thus, fictional narratives are neither wholly dependent on a pre-existing theory of mind nor the means by which we gain a theory of mind in the first place; instead, they exist along a continuum of which these two positions are merely the extreme points.

A final question emerges in relation to the distinction that motivated our work in the first place—that between enactivist and inferentialist models of social cognition. In this regard, our results argue for a pluralist position—like that advocated, for instance, by Leonhard Schilbach and colleagues—that recognises that sociality is not a discrete skill, but is instead composed of several competences that are deployed as need arises [3], [61]. Sometimes, this will take the form of explicit theorising concerning the motives of third parties; in others, gesture, facial expression, bodily orientation and behaviour will be pre-reflectively accepted as giving unmediated access to another's mental states, and acted on accordingly. Moreover, we expect that developmental stage will play a key role in determining which socialising competences are deployed, given that mindreading is one of the last skills to crystallise in ontogeny and is marked by distinct phases [62], [63]. The general point is that premature (or polemical) claims that social cognition is exclusively enactivist or inferentialist only serve to close off lines of inquiry that may well prove fruitful in the longer run.

When it comes to future work on this topic, we have already argued for the value of partitioning our results further in terms of the abilities—whether native or acquired—on the part of the experimental subjects. This, we expect, should not alone resolve the anomalous position of language complexity, but should also identify the traits that predispose readers to specific genres. We anticipate, however, that the most worthwhile line of future inquiry will pursue the issue of cognitive load. Here, we focused on the issue of ToM, as it is central to current debates in narrative scholarship. Nevertheless, mentalizing level is only one of the many ways in which a text can impose a cognitive load—just as infidelity detection strategies are only one of the many evolutionary inheritances that human beings are heir to. Correspondingly, any thorough account of how narrative cognition operates can only be achieved by way of a rigorous experimental programme that systematically plots the interplay of cognitive load, evolutionary cues and interpersonal variation—a research programme that we hope to follow up on in the future.

To close, we volunteer our results as an important contribution to our understanding of how the human mind utilises narrative. As has been recognised for some time now, no account of the mind can be considered complete without a recognition of the role played by narrative in the operations of thought—just as, equivalently, no assessment of narrative can afford to neglect our knowledge of cognition [64]–[69]. What is less clear, however, is how narrative and cognition interact, and the scope of this interaction when it occurs. Our results at least partially remedy this deficiency by clearly delineating the role played by narratives of different types in mediating our thinking about other minds. On the one hand, this contributes to the psychological project of understanding how cognition and behaviour interact; on the other, it gives us traction on theoretical questions in the humanities that have, up to now, defied easy resolution. Thus, considered in sum, the results articulated here form part of an interdisciplinary research agenda that points towards the basic unity of different forms of knowledge. For now, however, our aim will have been achieved if we have managed to make a useful intervention in in the question of whether fictional narratives presuppose or inform our reasoning about other minds.
